# Temporary spontaneous regression of male breast cancer: a case report

**DOI:** 10.1186/s40792-020-01088-1

**Published:** 2020-12-07

**Authors:** Kaoru Katano, Yutaka Yoshimitsu, Takahiro Kyuno, Yusuke Haba, Tsutomu Maeda, Seiko Kitamura

**Affiliations:** 1 Department of Surgery, Houju Memorial Hospital, 11-71 Midorigaoka, Nomi, Ishikawa 923-1226 Japan; 2 Department of Pathology, Houju Memorial Hospital, 11-71 Midorigaoka, Nomi, Ishikawa 923-1226 Japan

**Keywords:** Spontaneous regression, Breast cancer, Cribriform, Reappearance

## Abstract

**Background:**

Spontaneous regression (SR) of a malignant tumor is the partial or complete disappearance of primary or metastatic tumor tissue in the absence of treatment, which can be temporary or permanent. Here, we report an extremely rare case of male breast cancer that exhibited temporary SR followed by reappearance 8 months after tumor disappearance.

**Case presentation:**

A 70-year-old man presented at our hospital with a primary complaint of pain and a lump in his left breast. Ultrasonography revealed a hypoechoic lesion measuring 12 mm × 10 mm × 8 mm. Fine-needle aspiration cytology revealed numerous necrotic and degenerated cells and few sheet-like clusters of atypical ductal epithelial cells. The atypical cells had mildly enlarged nuclei with nucleoli, were focally overlapped and formed tubular patterns. The cytological diagnosis indicated a suspicion of malignancy. Core needle biopsy (CNB) revealed necrotic and degenerated cells with microcalcification. The pathological diagnosis was indeterminate because there was no area of viable atypical cells. An excisional biopsy of the left breast lesion was scheduled one month later. However, it was difficult to detect the tumor during physical examination and ultrasonography performed 1 month after the patient’s first visit. The operation was canceled, and the patient received follow-up observation. After 8 months of follow-up, ultrasonography and computed tomography (CT) revealed reappearance of a 0.6-cm-diameter breast tumor in the same place. CNB was performed again and revealed invasive ductal carcinoma. A total mastectomy with sentinel lymph node biopsy was performed 13 months after the first tumor disappeared. Histopathological examination revealed invasive cribriform carcinoma without sentinel lymph node metastasis. The patient did not have any complications, and adjuvant therapy with tamoxifen was started. The patient was alive without recurrence 7 months after surgery.

**Conclusions:**

Temporary SR followed by tumor reappearance can occur in breast cancer cases, and it is important to follow patients even if their breast tumor has seemingly disappeared. When breast tumors disappear without treatment, clinicians must be aware of the possibility of SR of cancer and should follow the patient for early detection of tumor reappearance.

## Background

Spontaneous regression (SR) of a malignant tumor is defined as “the partial or complete, temporary or permanent disappearance of primary or metastatic tumor tissue in the absence of treatment” [1, 2]. Immune mechanisms are the most commonly mentioned possible cause of SR; however, hormones, infections, operative trauma-related factors, and ischemia/necrosis-related factors have all been mentioned as possible causes of SR in the literature [3]. Although breast cancer is the most frequent cancer among women, there are few reports of SR of breast cancer. Here, we report an extremely rare case of male breast cancer that exhibited temporary SR followed by reappearance 8 months after tumor disappearance.

## Case presentation

**Fig. 1:**
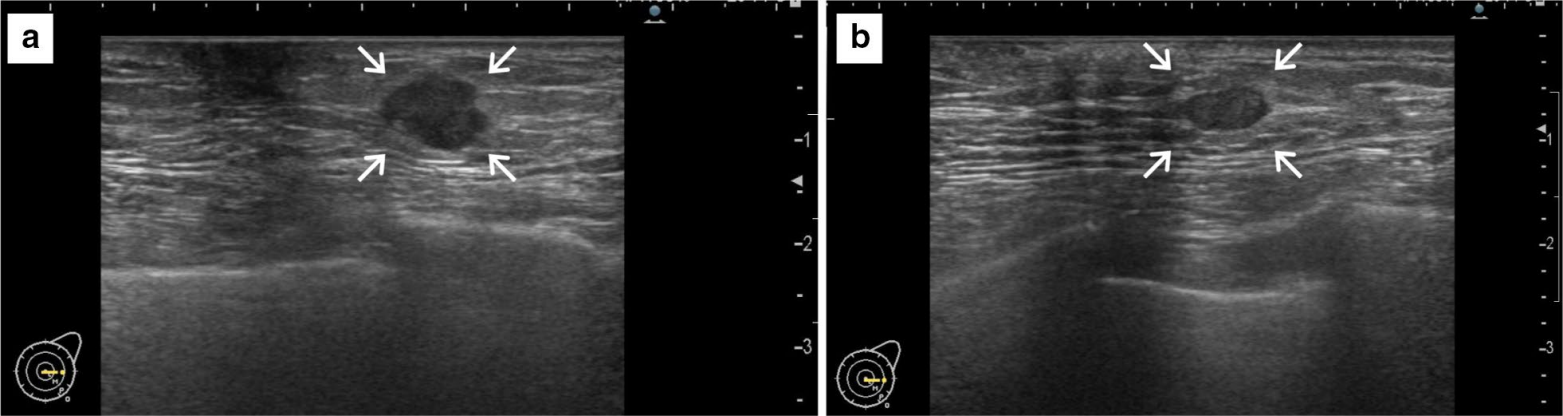
Ultrasonography findings. **a** On the patient’s first visit to our hospital, we observed a well-circumscribed, lobulated and hypoechoic lesion measuring 12 mm × 10 mm × 8 mm. **b** A week after the patient’s first visit, we observed a reduction in tumor size to 8 mm × 7 mm × 4.5 mm

**Fig. 2:**
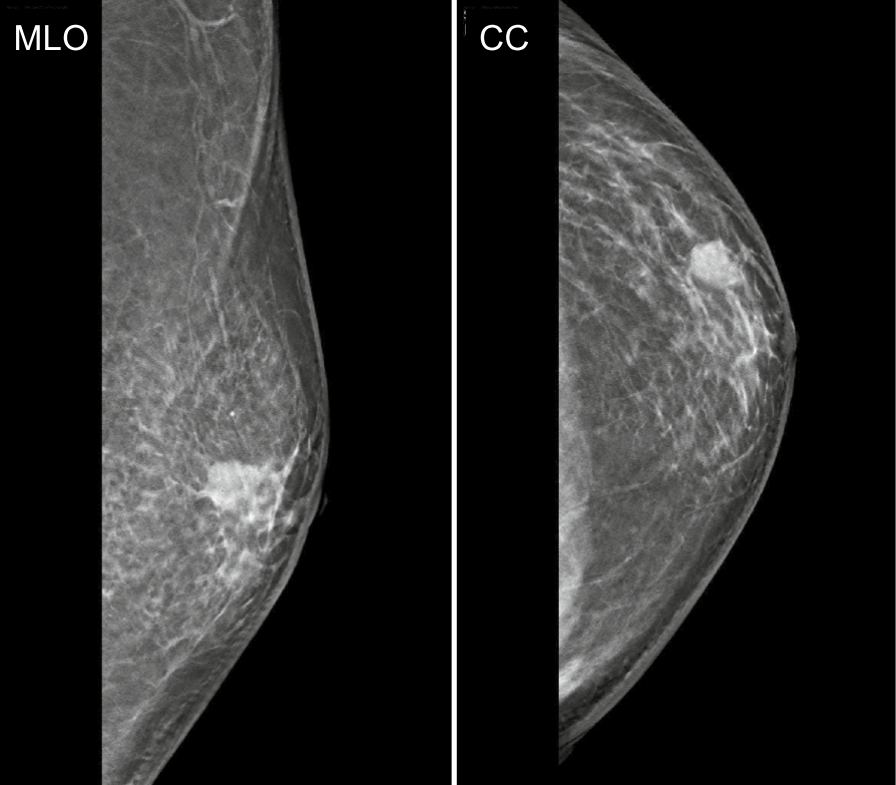
Mammographic findings showing a well-defined mass in the patient’s left breast. Note that MLO denotes medio-lateral-oblique and CC denotes cranio-caudal

**Fig. 3:**
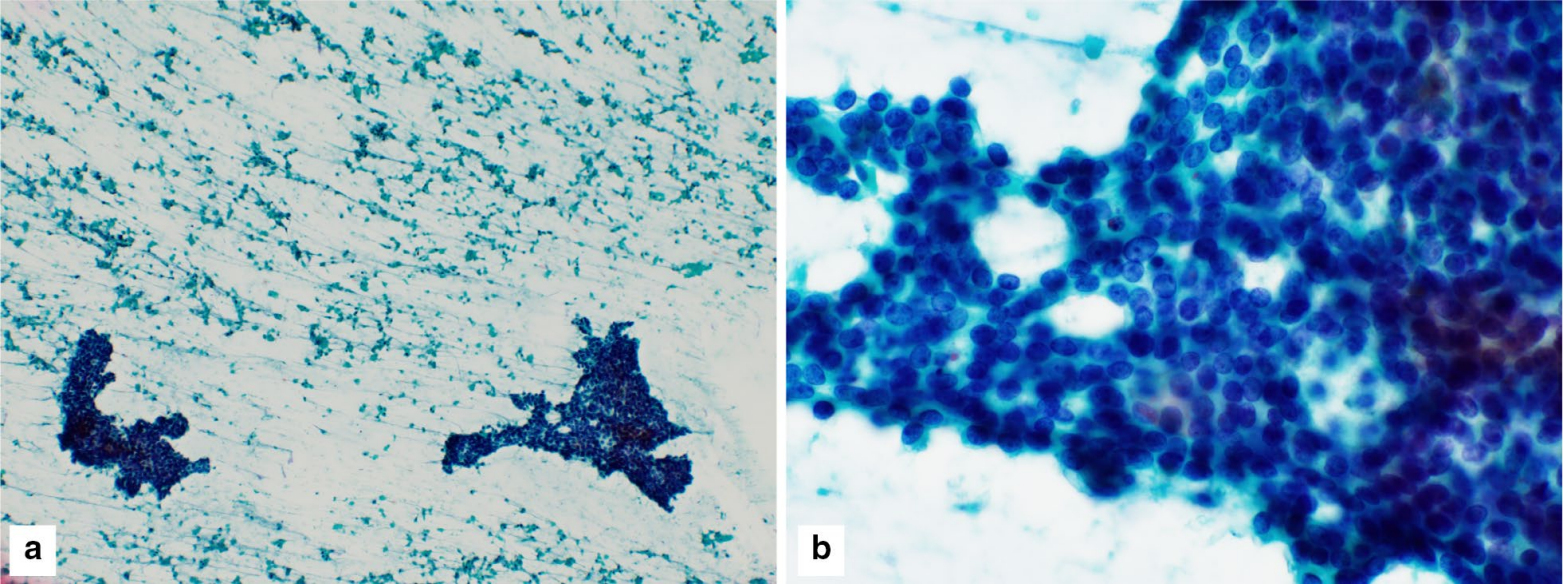
FNAC findings. **a** Numerous necrotic and degenerated cells and few clusters of atypical epithelial cells were observed. **b** The atypical epithelial cells had mildly enlarged nuclei with nucleoli, were focally overlapped and formed tubular patterns (Papanicolaou staining, **a** ×100, **b** ×600)

**Fig. 4:**
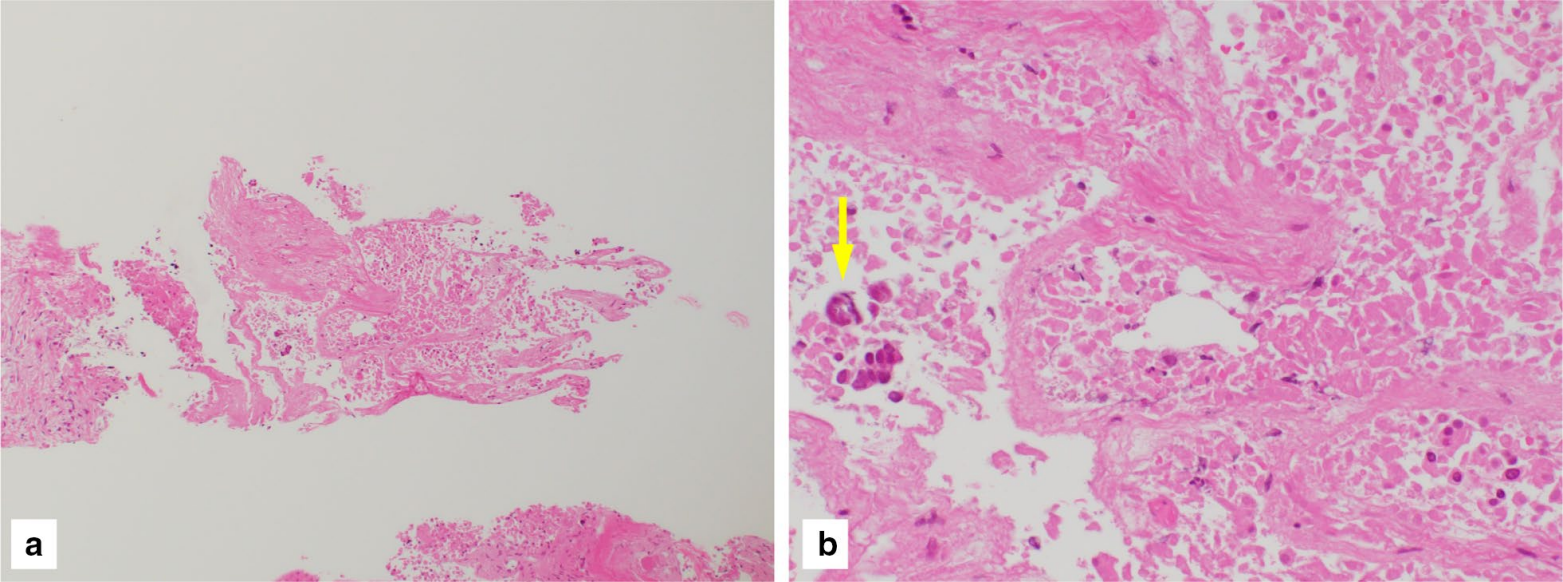
CNB findings. **a** A necrotic and degenerated area was observed. **b** Necrotic and degenerated cells with microcalcification (arrow) were observed (H&E staining, **a** ×100, **b** ×400)

**Fig. 5:**
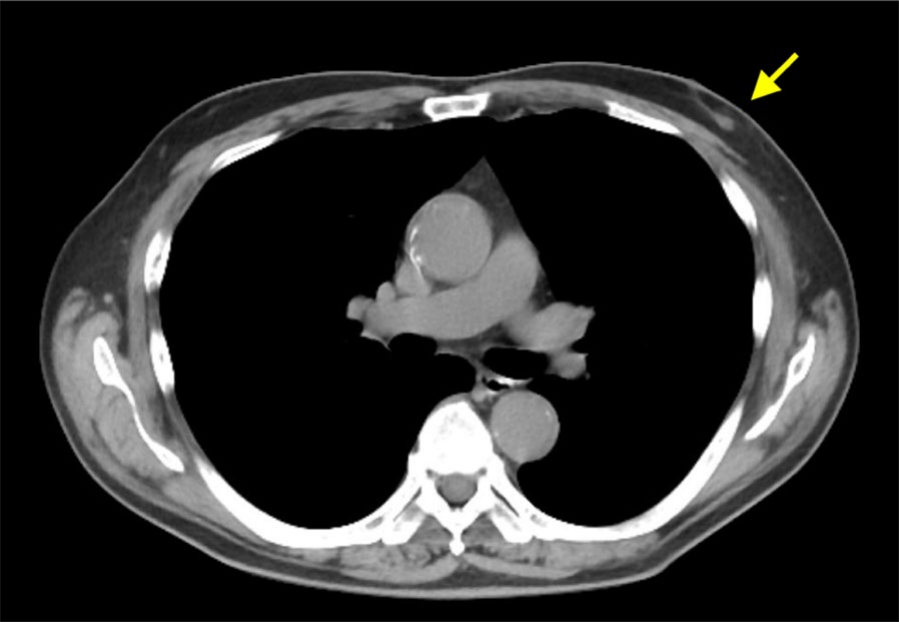
CT revealed a mass lesion with a linear shadow between the mass and nipple in the patient’s left breast

**Fig. 6:**
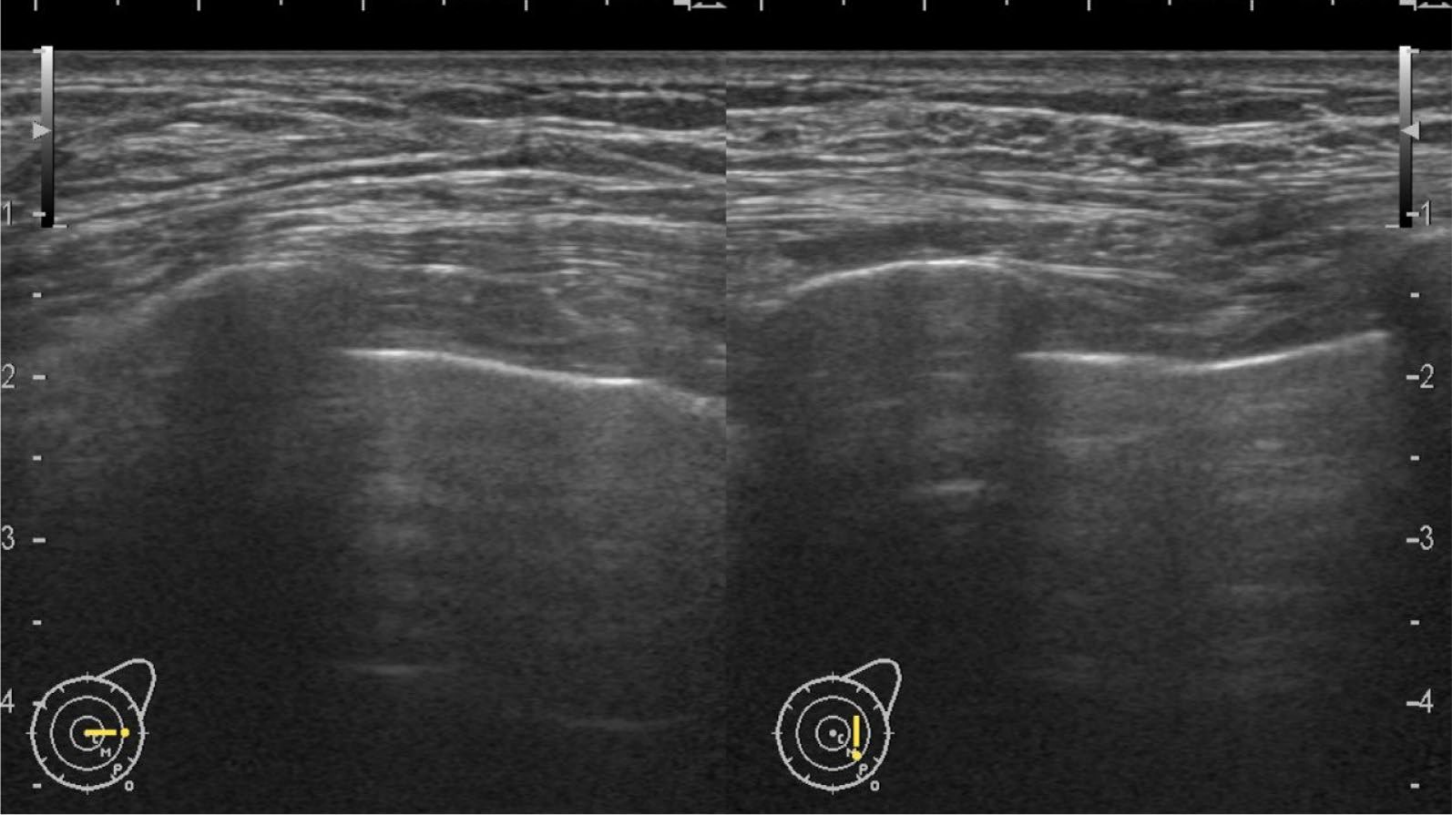
A month after the patient’s first visit, we could not detect the tumor on ultrasonography

**Fig. 7:**
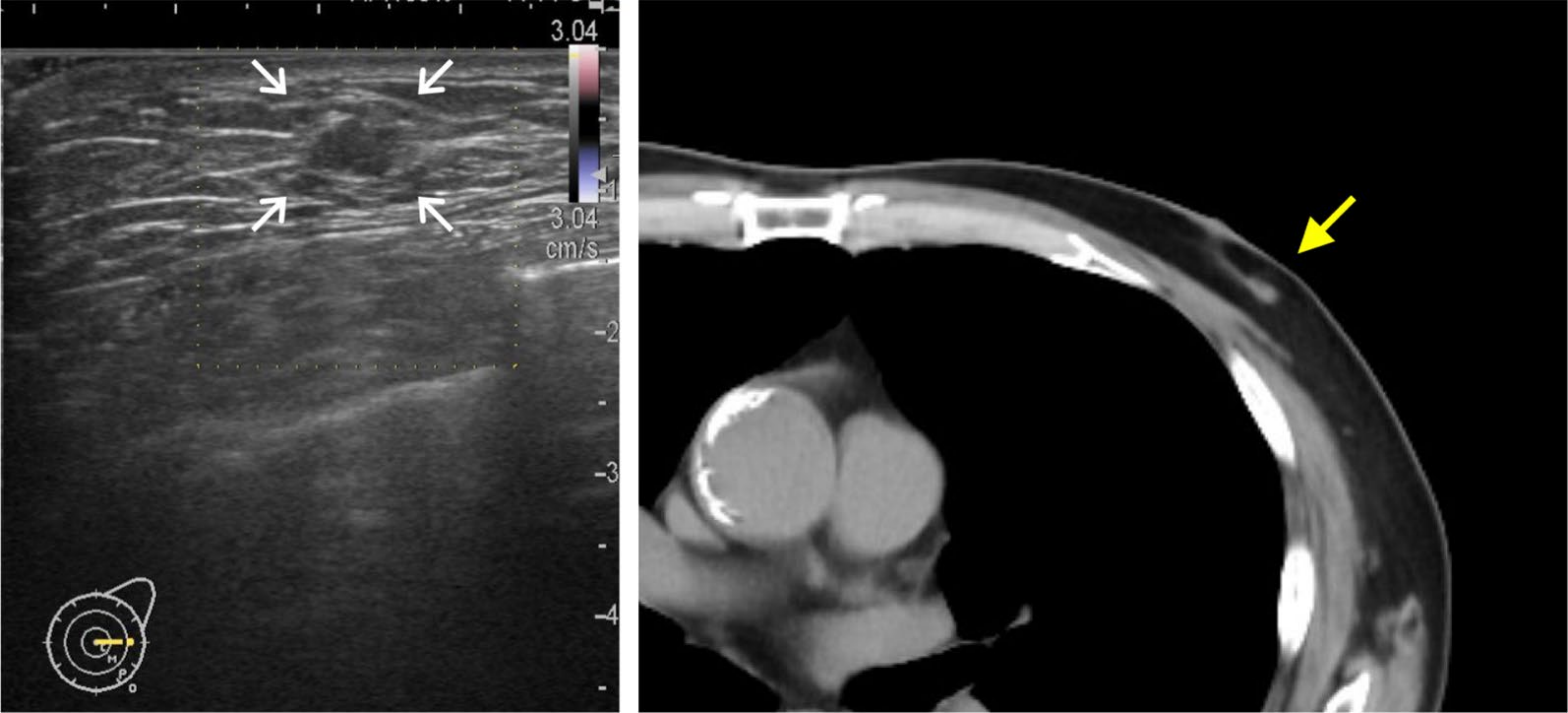
Imaging findings at 8 months after the disappearance of the tumor. Ultrasonography and CT revealed the reappearance of a 0.6-cm-diameter breast tumor in the same place as the previous mass

**Fig. 8:**
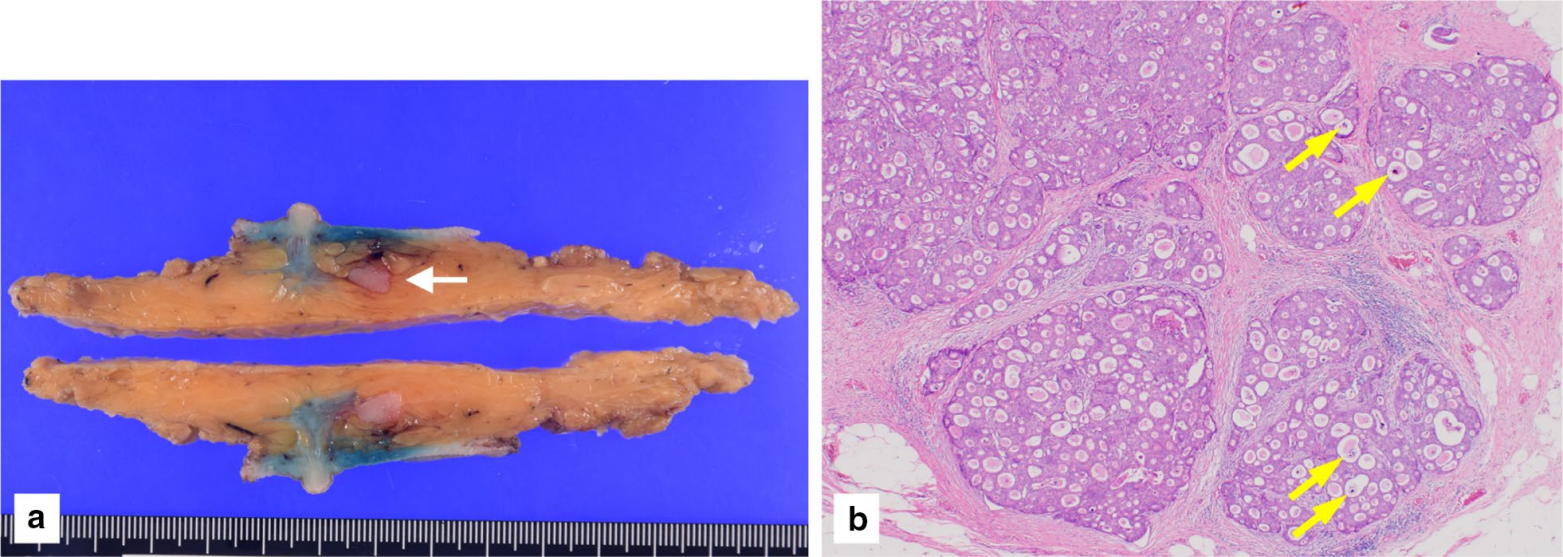
Pathological findings for a resected specimen. **a** Macroscopically, a gray-white, well-circumscribed mass with a diameter of 1.2 × 0.8 cm was observed (white arrow). **b** Histopathological examination revealed ICC with microcalcification (yellow arrows). Inflammatory cell infiltration was mild and focal (H&E staining, ×40)

A 70-year-old man with hypertension and type 2 diabetes presented at our hospital with a primary complaint of pain and a lump in his left breast. A physical examination revealed a 1-cm-diameter mass located on the border between the upper-outer and lower-outer quadrants of his left breast. A blood test revealed no abnormalities. The values of tumor markers were within normal limits (CEA, 2.3 ng/ml; CA15-3, 22.7 U/ml). Ultrasonography revealed a well-circumscribed, lobulated and hypoechoic lesion measuring 12 mm × 10 mm × 8 mm (Fig. 1a). It was not difficult to detect the tumor with physical examination and ultrasonography because it was located shallow from the body surface. Mammographic findings revealed a well-defined mass in the left breast (Fig. 2). Fine-needle aspiration cytology (FNAC) and core needle biopsy (CNB) were performed the day after the patient’s first visit. FNAC revealed numerous necrotic and degenerated cells and few sheet-like clusters of atypical ductal epithelial cells. The atypical cells had mildly enlarged nuclei with nucleoli, were focally overlapped and formed tubular patterns (Fig. 3). The cytological diagnosis indicated a suspicion of malignancy. On the other hand, CNB revealed necrotic and degenerated cells with microcalcification and myxomatous fibrous tissue with slight inflammation (Fig. 4). Massive infiltration of inflammatory cells was not observed. The pathological diagnosis was indeterminate because there was no area of viable atypical cells. A week after the patient’s first visit, physical examination revealed slight tumor shrinkage. Ultrasonography was performed again and showed a reduction in tumor size to 8 mm × 7 mm × 4.5 mm (Fig. 1b). Computed tomography (CT) revealed a mass lesion with a linear shadow between the mass and nipple in the patient’s left breast, although there were no swollen lymph nodes (Fig. 5). An excisional biopsy of the left breast lesion was scheduled one month later. However, during preoperative systemic examination, the patient noticed that the tumor had decreased in size, and it was difficult to detect the tumor during physical examination and ultrasonography performed one month after the patient’s first visit (Fig. 6). The operation was canceled, and the patient received follow-up observation. After 8 months of follow-up, ultrasonography and CT revealed reappearance of a 0.6-cm-diameter breast tumor in the same place (Fig. 7). CNB was performed again and revealed invasive ductal carcinoma. A total mastectomy with sentinel lymph node biopsy was performed 13 months after the first tumor disappeared. Macroscopically, a gray-white, well-circumscribed mass with a diameter of 1.2 × 0.8 cm was observed in the resected specimen. Histopathological examination revealed invasive cribriform carcinoma (ICC) without sentinel lymph node metastasis. Necrotic tissue was not observed, and inflammatory cell infiltration was mild and focal (Fig. 8). Immunohistochemical studies showed ER-positive, PgR-positive, and HER2-negative patterns. Androgen receptor staining was also positive, and the Ki-67 labeling index was 14%. The patient did not have any complications, and adjuvant therapy with tamoxifen was started. The patient was alive without recurrence 7 months after surgery.

## Discussion

This case reveals two important clinical suggestions. Temporary SR followed by tumor reappearance can occur in breast cancer cases, and it is important to follow patients even if their breast tumor has seemingly disappeared.

First, temporary SR followed by tumor reappearance can occur in breast cancer cases. SR of malignant tumors is a rare but well-documented biological phenomenon [4]. This phenomenon occurs approximately once in every 60,000–100,000 cancer cases [5], and it has been reported mainly in kidney cancer, neuroblastoma, malignant melanoma, and choriocarcinoma [6]. On the other hand, SR of breast cancer is rare. In a review of SR among 741 cases of cancer, only 43 cases (5.8%) of breast cancer exhibited regression [3]. We found only seven case reports of SR of breast cancer in an English literature search of PubMed from 1994 to July 2020 using the search words “spontaneous regression”, “spontaneous remission”, “breast cancer” and “breast carcinoma” (Table 1) [4, 6–11]. To the best of our knowledge, this is the first report of temporary SR of breast cancer followed by reappearance. In addition, ICC is an extremely rare, unique type of invasive ductal carcinoma that was first described by Page et al. in 1983 [12]. ICC is characterized by a cribriform pattern in the majority of its invasive component [13]. The incidence of ICC is approximately 0.4% among all female invasive breast carcinoma cases [14], and only a few male patients have been previously reported [13, 15].

**Table 1:** Reported cases of spontaneous regression of breast cancer

References	Year	Age/sex	Suggested mechanism	Possible trigger	Regression	Pathologically verified SR
[7]	1994	46 F	Immunological response mediated by activated CD8 + T cells and NK cells	Treatment with dexamethasone	Regression of primary tumor and metastatic lesion	−
[8]	2008	68 F	Immunological and local inflammatory response	Arm injury Needle biopsy	Complete regression of primary tumor	+
[4]	2014	52 F	Immunological response	Unclear	Nearly complete regression of primary tumor and complete regression of metastatic lymph nodes	+
[9]	2016	44 F	Immunological response	Needle biopsy	Complete regression of primary tumor	+
[6]	2019	67 F	Immunological response	Unclear	Nearly complete regression of primary tumor and complete regression of metastatic lymph nodes	+
[10]	2019	72 F	Immunological response mediated by activated CD8 + T cells	Unclear	Complete regression of primary tumor and metastatic lymph nodes	+
[11]	2020	86 F	Immunological response mediated by activated CD8 + T cells	Unclear	Regression of metastatic skin lesions	−
Our case	2020	70 M	Ischemia/infarction	Unclear	Regression of primary tumor followed by reappearance	−

As shown in Table 1, the infiltration of inflammatory cells was observed in the biopsy and/or excised specimens in all the reported cases, and immunological reactions were mainly considered to be the mechanism of SR in breast cancer. On the other hand, in the present case, extensive necrotic tissue was observed by CNB performed the day after the patient’s first visit. Furthermore, unlike other reported cases, massive inflammatory cell infiltration was not observed in the CNB and resected specimens. Therefore, ischemia/infarction-related factors may be involved in the SR of our patient’s tumor. In addition, it has been reported that spontaneous infarction often occurs in benign breast lesions, including fibroadenoma, intraductal papilloma and lactating adenoma, and the most frequent clinical manifestation is a painful breast mass [16, 17]. Our patient’s painful breast tumor might have been due to infarction of the tumor, although the mechanism and trigger of the necrotic change were unclear.

In five of seven reported cases [4, 6, 8–10], complete or nearly complete disappearance of tumor cells in the primary tumor and/or metastatic lymph nodes with infiltration of inflammatory cells was observed on pathological examination of surgical specimens. On the other hand, in two of seven reported cases [7, 11], regression of the primary tumor and/or metastatic lesion was observed on visual examination or CT. Although our patient’s breast tumor also disappeared according to physical examination and ultrasonography, the tumor reappeared 8 months later in the same place. It is conceivable that surviving cancer cells repopulated the site after nearly completely disappearing. The reason for this incomplete disappearance followed by repopulation of our patient’s tumor cells could be attributed to the fact that SR of the tumor was caused by infarction, not by an immunological response, as in other reported cases.

The second clinical suggestion is that it is important to follow patients even if their breast tumor has seemingly disappeared. In the present case, we followed the patient after his tumor disappeared because male breast cancer was suspected from the FNAC results, and this follow-up led to early detection of tumor reappearance. Hence, if FNAC had not been performed at that time, the breast lesion might not have been suspected to be malignant due to tumor shrinkage and disappearance, and follow-up might be completed after examinations were performed several times. In particular, male patients may be less likely than female patients to receive follow-up for breast tumors because the incidence of male breast cancer is low and breast cancer screening for men is generally not performed. Recently, a case of SR of lung carcinoids that reappeared approximately 2 years after nearly complete disappearance was reported [18]. Long-term follow-up is required for the early detection of breast tumor reappearance, even in male patients.

## Conclusions

Temporary SR followed by tumor reappearance can occur in breast cancer cases, and it is important to follow patients even if their breast tumor has seemingly disappeared. When breast tumors disappear without treatment, clinicians must be aware of the possibility of SR of cancer and should follow the patient for early detection of tumor reappearance. As other authors have described in similar reports, an improved understanding of the exact mechanism of SR of cancer may lead to the development of cancer treatment and prevention methods [19, 20]. Further reports should be accumulated to elucidate SR-related mechanisms.

## Data Availability

All data generated during this study are included in this published article.

## Abbreviations

SR: Spontaneous regression
FNAC: Fine-needle aspiration cytology
CNB: Core needle biopsy
CT: Computed tomography
ICC: Invasive cribriform carcinoma
